# Deleterious Effect of NMDA Plus Kainate on the Inner Retinal Cells and Ganglion Cell Projection of the Mouse

**DOI:** 10.3390/ijms21051570

**Published:** 2020-02-25

**Authors:** Estrella Calvo, Santiago Milla-Navarro, Isabel Ortuño-Lizarán, Violeta Gómez-Vicente, Nicolás Cuenca, Pedro De la Villa, Francisco Germain

**Affiliations:** 1 Department of Systems Biology, University of Alcalá, 28871 Madrid, Spainpedro.villa@uah.es (P.D.l.V.); 2 Department of Physiology, Genetics and Microbiology, University of Alicante, 03690 Alicante, Spain; 3 Department of Optics, Pharmacology and Anatomy, University of Alicante, 03690 Alicante, Spain; vgvicente@ua.es; 4 Instituto Ramón y Cajal de Investigación Sanitaria (IRYCIS), 28034 Madrid, Spain

**Keywords:** NMDA, KA, glutamate, excitotoxicity, Cholera toxin, ganglion cells

## Abstract

Combined administration of N-Methyl-D-Aspartate (NMDA) and kainic acid (KA) on the inner retina was studied as a model of excitotoxicity. The right eye of C57BL6J mice was injected with 1 µL of PBS containing NMDA 30 mM and KA 10 mM. Only PBS was injected in the left eye. One week after intraocular injection, electroretinogram recordings and immunohistochemistry were performed on both eyes. Retinal ganglion cell (RGC) projections were studied by fluorescent-cholerotoxin anterograde labeling. A clear decrease of the retinal “b” wave amplitude, both in scotopic and photopic conditions, was observed in the eyes injected with NMDA/KA. No significant effect on the “a” wave amplitude was observed, indicating the preservation of photoreceptors. Immunocytochemical labeling showed no effects on the outer nuclear layer, but a significant thinning on the inner retinal layers, thus indicating that NMDA and KA induce a deleterious effect on bipolar, amacrine and ganglion cells. Anterograde tracing of the visual pathway after NMDA and KA injection showed the absence of RGC projections to the contralateral superior colliculus and lateral geniculate nucleus. We conclude that glutamate receptor agonists, NMDA and KA, induce a deleterious effect of the inner retina when injected together into the vitreous chamber.

## 1. Introduction

It has been shown that the toxicity caused by glutamate analogues is at the center of many neuronal degenerative diseases. An important example is cerebral ischemia—since just ten minutes of acute hypoxia can cause brain damage, which continues to increase for months [1], even if vascular flow is recovered. The cause of this, and of many other neurodegenerative processes, seems to be the large amount of excitatory neurotransmitter glutamate released and the large number of receptors it stimulates.

In recent years, multiple animal models of neuronal degeneration have been studied. Those that affect the retinal ganglion cells (RGCs), such as retinal ischemia, optic nerve injury, glaucomatous optic neuropathy or excitotoxic processes [2], are especially interesting from a pathophysiological point of view. This is due in part to the easy access to the retina which allows an accurate count of the RGCs to be made, and to carry out a functional and histological analysis of different retinal cells. Ganglion cells collect information from second-order neurons in the retina, and transmit it to higher centers through the optic nerve. It has been reported that the loss of RGCs, or just their functional lesion, may affect the transmission of the light signal to higher centers of the visual pathway. Frequently, the administration of glutamate receptor antagonists provides protection of these retinal cells, suggesting that a toxic effect of glutamate triggered by different etiologies might be involved [3,4,5,6]. 

The use of glutamate agonists such as kainic acid (KA) or N-methyl-D-aspartic acid (NMDA) has been developed to induce a selective damage on the RGCs [7,8]. Several studies, both in rat and mouse, have been described and all agree that the intraocular injection of NMDA and / or KA causes a loss of RGCs [7,9,10,11,12,13]. This cell loss is dependent on the inoculated dose of NMDA, since, while a dose of 10 nmol did not cause any cell loss, the intraocular injection of 160 nmol may have induced a cell loss of ca. 70% [6], resembling these models to those obtained by crushing the optic nerve [14,15]. 

Previous functional evaluation of the rat retina by electroretinogram (ERG) recordings, showed that 3 days after the intraocular injection of NMDA, a reduction in the amplitudes of the positive and negative waves of the scotopic threshold response (STR), an attenuation of the scotopic “a” and “b” waves, and a decrease in the oscillatory potentials (OP) wave amplitude may be observed [16], in parallel to the decrease in the number of RGCs. 

On the other hand, histological studies showed that the cell death process included condensed pyknotic nuclei and DNA fragmentation, both characteristics of apoptotic cell death. However, the way in which the cell death process occurred seems to depend on the magnitude of the excitatory response generated by the inoculated dose [3]. 

Another factor that influences sensitivity to excitotoxic agents is the type of RGC. Thus, the Alpha RGCs are more resistant to excitotoxicity by NMDA, while other RGCs are more sensitive, which suggests the importance of cell specific neuroprotective mechanisms [17]. 

In addition, some evidence from different neural tissues shows that glutamate could act on different subpopulations of NMDA receptors to generate different results. For example, NMDA receptors stimulated at low doses promote neuronal survival in granular cells of the cerebellum in culture [18,19]. However, at high doses they promote neuronal death [20,21]. Therefore, depending on the level of activity of NMDA receptors, survival or neuronal death occurs. Also, the specific localization of the proteins that are activated by NMDA would have a fundamental role, so that the synaptic receptors (containing the GluN2A subunit) promote survival; while the extra-synaptic receptors (containing the GluN2B subunit) would be activated when there is much glutamate in the brain during cerebral ischemia, inducing neuronal death [22].

Finally, the main aim of the present work was to verify that the combination of two drugs, agonists of different glutamate receptors, may mimic the real effect of glutamate as the most important excitatory agent and cause a deleterious effect on cells of the inner retina. This severe damage may be addressed by functional tests or structural evaluation of the retina, and its projections to centers of the visual pathway.

## 2. Results

### 2.1. Dose Adjustment of Excitotoxic Agents

In a preliminary series of experiments, the damaging effect of different concentrations of glutamatergic agonists on retinal ganglion cells was evaluated. Surviving RGC counts were performed 7 days after the administration of individual doses of KA and NMDA. It was observed that the minimum dose of KA that causes a statistically significant difference between the damaged and control eye was 10 mM, while the minimum dose of NMDA capable of evoking some visible damage was 30 mM. Table 1 shows the data related to the RGC count experiments. 

Based on these results, various combinations of NMDA and KA were selected, and the excitotoxicity experiments on ganglion cells were repeated. The combinations used were: 10 mM NMDA and 5 mM KA; 10 mM NMDA and 10 mM KA; 15 mM NMDA and 5 mM KA; 30 mM NMDA and 5 mM KA; 30 mM NMDA and 10 mM KA. Cell counts showed a loss of RGC of 12%, 15%, 55%, 63% and 75%, respectively. The joint dose of 30 mM NMDA and 10 mM KA was thus selected, as it was the one that induced a more significant loss of RGC. Table 1 also collects the cell count data obtained after the co-administration of NMDA and KA.

### 2.2. Functional Analyses 

**Figure 1:**
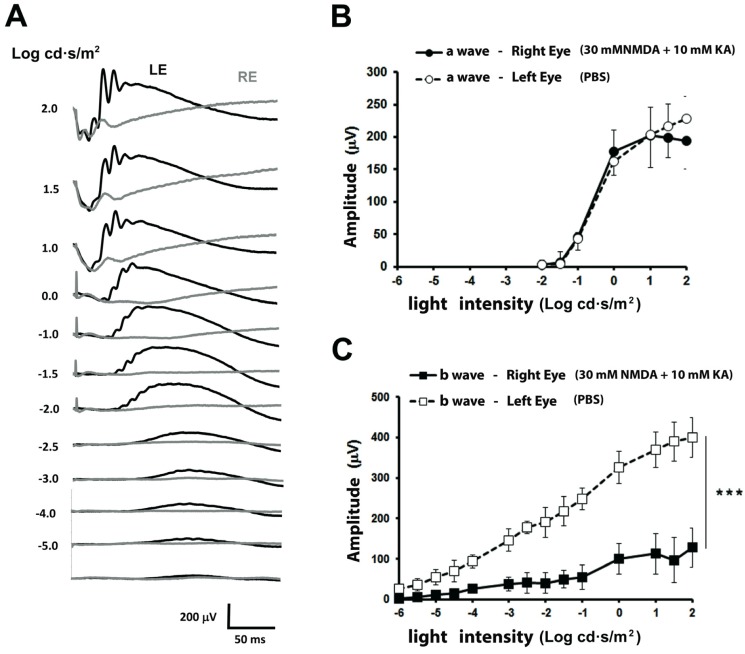
Scotopic electroretinographic responses after intraocular injection of NMDA and Kainate. (**A**) Electroretinographic recordings obtained from the right eye injected with 30 mM NMDA and 10 mM KA (RE, gray trace) and left eye injected with PBS (LE, control, black trace) in response to increasing light intensities (shown to the left of each record in Log cd·s/m^2^). (**B**) Relationship between the amplitude of the a-wave and the light intensity showing no differences between eyes. (**C**) Relationship between the amplitude of the scotopic b-wave and the light intensity. The values shown in (**B**,**C**) correspond to the mean and the standard deviation, *n* = 8.

The functional responses to the intraocular injection of 30 mM NMDA and 10 mM KA were studied and compared with those from vehicle intraocular injection in the contralateral eye 7 days after inducing the damage (Figure 1). The electroretinographic recordings were initially performed under dark adaptation. Light intensities of −5.0 to −2.5 log cd·seg/m² evoked a positive response (“b” wave) in the control eye, while the amplitude of the b-wave recorded in the NMDA/KA injected eye was almost absent for these light intensities. From −1.5 to 2 log cd·seg/m² increasing intensities, a negative response (“a” wave) was recorded from both eyes, without showing significant differences between eyes. Increasing light intensities applied on the left eye evoked b-wave responses of increased magnitude, while in the right eye the b-wave response increase was significantly smaller (Figure 1A). The relationship between a- and b-wave amplitudes and the intensity of illumination is shown in Figure 1B,C. The a-wave amplitudes of both eyes grow as light intensity is increased (see Figure 1B), showing no differences between eyes. However, the amplitude of the b-wave of the eye injected with NMDA/KA showed clearly, the smaller amplitude than the eye injected with PBS (Figure 1C). 

**Figure 2:**
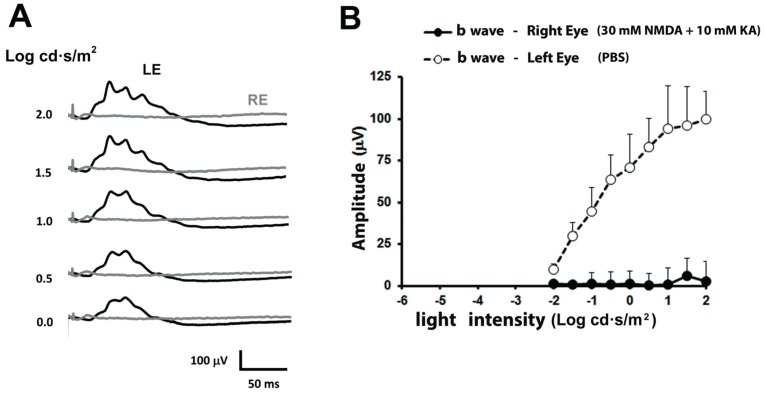
Photopic electroretinographic responses after intraocular injection of NMDA and Kainate. (**A**) Electroretinographic records obtained in the right eye injected with 30 mM NMDA and 10 mM KA (grey trace) and left eye injected with PBS (black trace) of the same animal shown in Figure 1, under increasing light intensities. (**B**) Relationship between the amplitude of the photopic b-wave and the light intensity. The values shown in (**B**) correspond to the mean and the standard deviation, *n* = 8.

Under photopic conditions, the electroretinographic response of the left eye showed, for luminous intensities of 0 to 2 log cd·seg/m², a photopic b-wave of increasing amplitude (Figure 2A). However, the b-wave was almost null in the right eye for any of the applied light intensities. The relationship between photopic b-wave amplitudes and the intensity of illumination is shown in Figure 2B. From these experiments, a clear functional damage of the retina is demonstrated.

**Figure 3:**
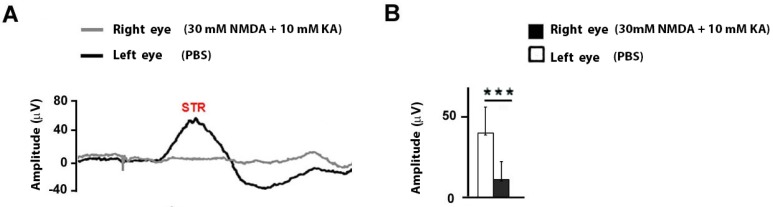
Amplitude of the STR wave after intraocular injection of NMDA and Kainate. (**A**) Electroretinographic records of the STR wave obtained in the right eye injected with 30 mM NMDA and 10 mM KA (gray trace) and left eye injected with PBS (black trace) in response to −4,5 log cd·s/m^2^. (**B**) Comparison of the STR wave amplitude between both eyes. The values shown in (**B**) correspond to the mean and the standard deviation, *n* = 11.

The electroretinographic response of the inner retina was further studied by analyzing the scotopic threshold response (STR) under scotopic conditions, and recorded in response to dim light intensities (−4,5 log cd·s/m^2^). The response of the STR wave showed a small amplitude in the eye injected with NMDA/KA (11.11 ± 11.18 μV), while the eye inoculated with the vehicle showed normal amplitudes (40.17 ± 16.45 μV). The statistical analysis between these two responses (Student’s *t* test) showed extremely significant differences (*p* ˂ 0.0001) (see Figure 3A,B).

**Figure 4:**
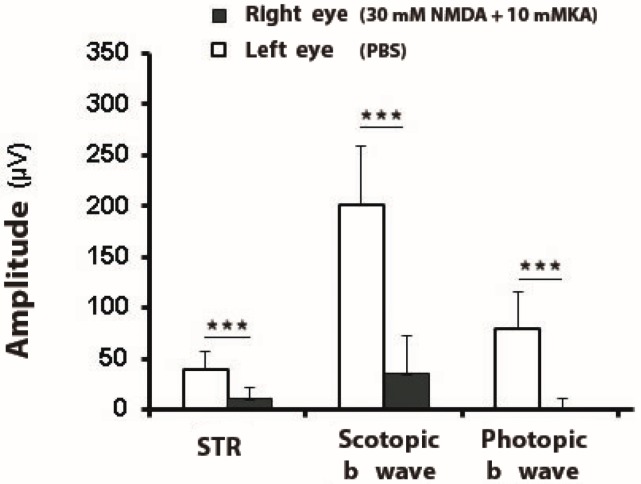
Amplitudes of electroretinographic waves after intraocular injection of NMDA and Kainate. The amplitude (mean and SD) of the scotopic threshold response STR (−4,5 log cd·s/m^2^), scotopic b-wave (−2 log cd·s/m^2^), and photopic b-waves (2 log cd·s/m^2^) were compared in a group of mice subjected to intraocular injection with 30 mM NMDA and 10 mM KA in the right eye (RE) versus the values obtained in the left eye injected only with PBS (LE, control eye) (***, extremely significant difference, *p* < 0.001), *n* = 11.

In summary, the ERG recordings of the animals exposed to the excitotoxic injection of NMDA/KA showed extremely significant amplitude differences with the control eye in the scotopic and photopic conditions, mainly related to the b-wave amplitudes, as well as STR wave response (Figure 4). 

### 2.3. Histological Analysis

**Figure 5:**
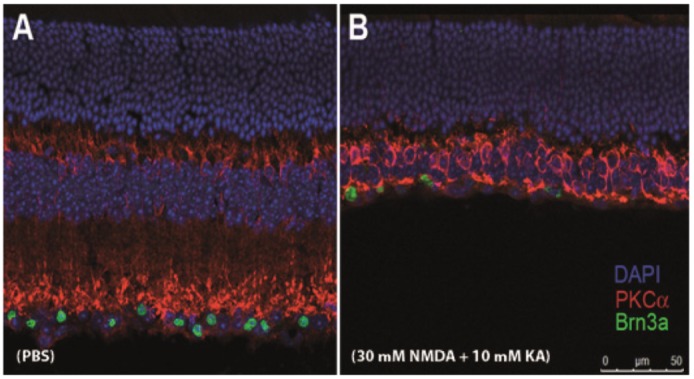
Cross retinal sections in a mouse eye after intraocular injection of NMDA and Kainate. (**A**) Left eye; control eye injected with PBS. (**B**) Right eye injected with 30 mM NMDA and 10 mM KA. Cells were labeled with DAPI (nuclear marker), PKCα (rod bipolar cell marker) and Brn3a (ganglion cell marker) seven days after intraocular injection.

The functional data suggest that the injection of NMDA plus KA induces both scotopic and photopic damage. The structural comparison of the injected and non injected retinas, by means of immunohistochemistry with antibodies that label rod bipolar cells and ganglion cells (PKCα and Brn3a, respectively), allowed to observe the excitotoxic effects generated in the different retinal layers. The NMDA/KA injected eye suffered significant structural damage, since a decrease in cell number and clear thinning of retinal layers was observed. A huge effect of the excitotoxic agents was observed on the inner nuclear layer, the inner plexiform layer, and the ganglion cell layer, while the photoreceptor layer did not appear to be altered. The layers of the eye injected with NMDA/KA appeared unstructured (Figure 5). However, it has also been observed that the inner nuclear layer (INL) maintains a certain number of rod bipolar cells (labeled with PKCα), which justifies some ERG responses under scotopic conditions.

**Figure 6:**
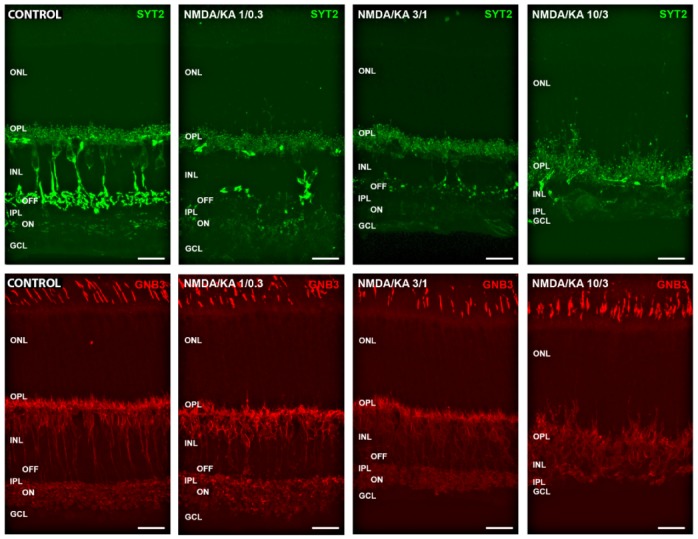
Effect of different NMDA/KA doses on retinal interneurons. Cone bipolar cells were labeled with Syt2 (OFF bipolar cells, green) and GNB3 (ON bipolar cells, red). The axon terminals of both cell types were clearly labeled at the OFF and ON sublayers of the inner plexiform layer in control conditions. The pictures show a progressive degradation of both types of bipolar cells under the effect of increasing concentrations of NMDA/KA (1/0.3–3/1–10/3), although some labeling remains even at the highest doses. ONL, outer nuclear layer; OPL, outer plexiform layer; INL, inner nuclear layer; IPL, inner plexiform layer; GCL, ganglion cell layer. Calibration bar: 20 µm.

Given that the dose of 30 mM NMDA and 10 mM KA produced a highly deleterious effect, not only on RGC, but also on retinal interneurons, a series of trials were proposed using gradually lower concentrations of NMDA and KA (10: 3; 3: 1; 1: 0.3 and 0.3: 0.1) in order to observe its effect on different types of retinal bipolar cells. Specific immunocytochemical labels were made for bipolar cells of the OFF type (labeled with antibodies against Syt2) and of the ON type (marked with antibodies against GNB3) (see Figure 6). The images show a gradual degeneration of these cells as the NMDA/KA doses increase, which is not yet complete for concentrations of 10 mM NMDA and 3 mM KA.

### 2.4. Visual Pathway Analyses

**Figure 7:**
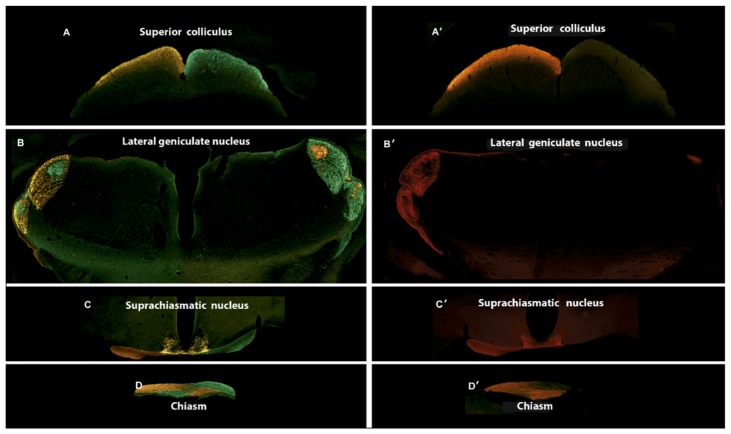
Central projections of retinal ganglion cells after intraocular injection of NMDA and Kainate. The Alexa Fluor® 488 and Alexa Fluor® 555 conjugates of the B subunit of the cholera toxin were injected intraocularly, into the left and right eyes, respectively, nine days after NMDA and Kainate injection. (**A**–**D**) Central projections in control animal. (**A’**–**D’**) Central projections in an experimental animal previously injected with 30 mM NMDA and 10 mM KA into the right eye (**A**,**A’**) Section at the level of the superior colicullus (−3.52 mm from Bregma). (**B**,**B’**) Section at the level of the lateral geniculate nucleus (−2.18 mm from Bregma). (**C**,**C’**) Section at the level of the suprachiasmatic nucleus (−0.34 mm from Bregma). (**D**,**D’**) Section at the level of the optic chiasm.

It was observed that the injection of excitotoxic agents in the eyeball caused the death of the ganglion cells. To check if any of the surviving RGCs was able to project to higher centers of the visual pathway, nine days after the injection of NMDA/KA Alexa Fluor® 488 and Alexa Fluor® 555 conjugates of the B subunit of the cholera toxin were injected intraocularly, respectively, into the left and right eyes. When these tracers are injected into the eyes of healthy animals, the fluorochrome marks the different projections and nuclei of the visual pathway that come from that eye, so that all projections emerging from the left eye were marked in red and all projections emerging from the right eye were marked in green. Figure 7 shows the coronal brain sections of the injected animals that represent four different brain sections along the visual pathway, which correspond to sections at the level of the optic chiasm (OC), suprachiasmatic nucleus (SN), lateral geniculate nucleus (LGN) and superior colliculus (SC). After intraocular injection, the cholera toxin that was picked up by the ganglion cell advances to the optic chiasm (Figure 7D), where a large portion (>90%, in mouse) of the axons decussate to the other side of the brain. The suprachiasmatic nuclei receive axons from RGCs of both eyes (Figure 7C). The projection of RGC axons determines the labeling of the lateral geniculate nuclei: The shell of the contralateral nucleus and the core of the ipsilateral one (Figure 7B); from there, the projection reaches the superior colliculi (Figure 7A). The use of both conjugates of the cholera toxins allowed the ipsilateral and contralateral projections of the visual pathway to be determined. 

After the selective injection in the right eye with the solution containing NMDA/KA, and subsequently the injection with Alexa Fluor® 488 conjugates of the B subunit of the cholera toxin, an absence of marking throughout the corresponding central projections may be observed (Figure 7A’–D’). However, when we injected the left eye with Alexa Fluor® 555 conjugates of the B subunit of the cholera toxin, a normal labeling of RGC projections may be observed along the visual pathway.

## 3. Discussion

The main functional finding observed after the intraocular injection of 30 mM NMDA and 10 mM KA into the eye was a significant decrease of the scotopic b-wave, and a significant decrease of the photopic b-wave. The functional study of the inner retina by analyzing the STR wave showed a significant decrease in its amplitude. In the structural analysis, a very significant decrease in the thickness of the internal retina (ganglion cell layer, inner plexiform and inner nuclear layers) was observed, although a significant number of rod bipolar cells remained present. The outer nuclear layer containing photoreceptors nuclei, which would be responsible for maintaining the scotopic a-wave, was also observed. On the other hand, the excitotoxicity of RGCs determines a deleterious effect and a complete fail of RGCs to project to higher visual centers, LGN and SC. 

The functional observation of maintained a-wave allows confirmation that the injection of NMDA/KA excitotoxic agents did not produce a direct damage of the photoreceptors. A previous study showed that the a-wave decreases after intraocular injection of excitotoxic agents [16]. In the study, the records of the b-wave showed a diminished response in the eye inoculated with NMDA/KA, which was more pronounced in photopic conditions, such results coincide with ours. On the other hand, immunohistochemistry showed the presence of rod bipolar cells; this may be justified by the absence of NMDA or KA receptors in these cells [23]. However, under photopic conditions the response of b-wave was absent, indicating that cone bipolar cells are especially affected by excitotoxicity.

The function of the ganglion cells was studied by analyzing and comparing STR in both eyes under scotopic conditions. The response of the STR wave is obtained by applying a very dim stimulus on a retina completely adapted to the darkness. It is thought to reflect the activity of the RGCs [24]. This electrophysiological record is considered a good measure of the function of the RGCs [25,26]. The scotopic threshold response is constituted by two waves (positive and negative), being the relationship between the positive STR and the number of RGC quite accepted. A significant reduction of the STR wave has been associated with eye diseases that affect the RGCs, such as ocular hypertension [27], as well as in models of hypertension in monkeys and glaucoma in humans [25,28].

Regarding the impairment of the internal retina, the RGCs are not the only cells that form this layer of the mouse retina, since 59% of the cells of this layer are displaced amacrine [29,30,31]. Thus, a selective RGC immunollabeling is necessary, such as what Brn3a does, which allows the number of surviving RGCs after the injury to be quantified [32,33]. These data allow us to ensure that the modifications, mainly observed in the ERG, are due to the neuronal loss of the internal retina, specifically the RGCs. The effect of excitotoxicity on the retinal thickness shown in our work coincides with those of Nakano et al. They observed that, 14 days after the intraocular injection of NMDA, a decrease in the thickness of the RGC layer was caused. It was observed that such a decrease in thickness began on day 4, although the decrease in the number of RGCs already began on day 1 and continued later [12]. It could be expected that some of the surviving RGCs would be able to keep their projections out of the retina. Indeed, it has been reported that melanopsin containing RGCs may survive retinal insults better than the rest of RGCs [34]. It has also been observed that these melanopsin cells not only survive for a longer-term, but also show signs of remodeling in their processes in response to retinal dystrophy [35]. Based on these findings, it has been speculated that they might maintain their connections with higher centers of the visual pathway. However, our results based on cholera toxin labeling show unequivocally that no labeling occurs outside the eye at any level of the visual pathway. Given the high percentage of RGC fibers that decussate in mice (90%), only a minimum marking was observed in the brain on the contralateral side of the NMDA/KA injected eye.

## 4. Material and Methods

### 4.1. Animal Model 

A total of 50 two-month old mice of the C57BL/6J strain were purchased from the Jackson Laboratory (Bar Harbor, ME, USA) as animal models. The animals were fed “ad libitum” with A04 from Panlab S.L.U. (Barcelona, Spain) and were maintained in a 12:12 h circadian cycle. The animals were treated according to the European (*Directiva* 86/609/EEC) and Spanish laws (*Real Decreto* 53/2013, of February 1, 2013). The University of Alcala research ethics and animal experimentation committee approved all protocols. In addition, the guidelines of the association for vision and ophthalmology research were followed (The Association for Research in Vision and Ophthalmology, ARVO). When necessary, the animals were sacrificed with a lethal overdose of a solution of 20% sodium pentobarbital (Dolethal®, Vetoquinol S.A., Lure, France) injected intraperitoneally (ip) (0.5–1 mL).

### 4.2. Intravitreal Injection of Excitotoxic Agents 

The animals were anesthetized by intraperitoneal injection of a mixture of ketamine (70 mg/Kg, Ketolar® 5% Pfizer, Alcobendas, Madrid, Spain) and xylazine (7 mg/Kg, Rompun® 2% Bayer, Kiel, Germany) in 0.1 ml sterile saline (NaCl, 0.9%). One microliter of a mixture of 30 mM NMDA (6384-92-5, Sigma-Aldrich, Darmstadt, Germany) and 10 mM KA (58002-62-3, Sigma-Aldrich) was injected into the right eye; while one microliter phosphate saline (PBS) was injected into the left eye. The intraocular injection process was performed under a microdissection microscope with cold light illumination source (Wild Heerbrugg, Intralux HE, Switzerland). One microliter-calibrated syringe (Nanofil Tm, World Precision Instruments, Sarasota, FL, USA) with a 35G needle (Nanofil Tm, NF35BV-2, World Precision Instruments) was used for intraocular injection. In the immediate postoperative period, 2% Methocel (Ciba Vision AG, 8442 Hetlingen, Switzerland) was applied topically to the cornea, to prevent corneal desiccation.

### 4.3. Dose Estimation of Excitotoxic Agents

Since the doses administered to induce retinal excitotoxicity injury vary according to the literature used [17,36,37,38,39,40,41], a series of experiments were performed to determine the ideal dose of joint administration of KA and NMDA in our model. Initially, in a series of mice (*n* = 3–4), different individual concentrations of NMDA (10, 30 and 100 mM) and KA (5, 10 and 20 mM) were administered by intraocular injection (see above), and the density of surviving RGC was estimated. The RE was used to inject the cytotoxic agent, while in the LE, PBS was injected as a control. The final joint concentration of 30 mM NMDA and 10 mM KA was chosen after trials of five different combinations of NMDA and KA (10:5; 10:10; 15: 5; 30: 5 and 30:10, in mM).

### 4.4. Retinal Ganglion Cell Density 

Previously, for eye enucleation, a mark was made on the superior pole of each eye to keep the retinal orientation. The anterior pole of the eye and vitreous body were carefully removed and the retina was dissected out. After fixation in freshly made 4% (*w*/*v*) paraformaldehyde in 0.1 M PBS (pH 7.4) for 1 h at room temperature, and washing several times with PBS, the retinas were flat mounted. The retinas were incubated for 72 h at 4 °C with primary goat anti-Brn3a antibody (1/200; sc-31984L, Santa Cruz Biotechnology Inc., Santa Cruz, CA, USA). Immunohistochemistry negative controls were conducted in parallel omitting the primary antibody. After several washes with PBS, the retinas were incubated for 2 h at room temperature with a Cy2 (FITC) donkey anti-goat secondary antibody (1/200, Jackson Immunoresearch Europe, Cambridge, UK). Then, the retinas were washed, and mounted on glass slides with the vitreous side up, cover-slipped with anti-fading mounting medium (Citifluor Ltd., London, UK) and sealed with nail polish.

The quantification of the RGC immunostained with Brn3a was performed on three standard areas (700 × 300 μm^2^) in each flat mounted retina. These include areas of high and low density and are representative of the total RGC population [42].

For the manual cell count, the Image J program (digital image processing program developed in 1997 by the National Institutes of Health, Bethesda, MD, USA) was used. With the image program, the bounded areas of the retina were selected where the RGC were stained with Brn3a to mark them manually and quantify them automatically.

The main utility of the manual counting method is that it allows microglia cells to be discarded. These cells, after RGC damage, are marked transcellularly and could distort the actual quantification of the RGC [43].

### 4.5. Electroretinographic Recordings 

A custom designated Ganzfeld hood was used to obtain the rod-mediated response, under dark adaptation (>12 h of continuous dark) with light intensities below 0.01 cd·s/m^2^. Mixed responses generated by cones and rods were recorded for light intensities greater than 0.01 cd·s/m^2^ under dark adaptation. After light adaptation (>5 min adaptation to a background light of 30 cd/m^2^), cone responses were recorded for light intensities greater to 0.01 cd·s/m^2^. 

Two corneal electrodes were used for ERG recording simultaneously from both eyes (Burian-Allen, Hansen Ophthalmic Development Lab, Coralville, IA, USA). A drop of 2% methyl-cellulose (Methocel) was instilled between corneas and electrodes. Mice were maintained for >10 min in absolute darkness before the recordings. Mouse temperature was maintained at 37 °C during the recording with a heating pad (Hot-Cold, Pelton Shepherd Industries, Stockton, CA, USA). Recorded electrophysiological responses were amplified, filtered (CP511 AC amplifier; Grass Instruments, Quincy, MA, USA), and digitalized (ADInstruments Ltd., Oxfordshire, UK). The recording process was controlled with Scope version 3.8.1 software (Power Lab, ADInstruments Ltd.).

### 4.6. Immunocytochemistry

Enucleation and anterior pole/vitreous removal was performed as described above. The whole eye was fixated in freshly made 4% (*w/v*) paraformaldehyde in 0.1M PBS (pH 7.4) for 1 h at room temperature and washed several times with PBS. After fixation, the eyeball was cryoprotected by serial baths at concentrations of 20, 30, 40% of sucrose diluted in 0.1M PBS. Subsequently, the eyes were included in a suitable medium for freezing (Optimal Cutting Temperature media, Sakura Finetek, CA 90501, USA) being stored at −80 °C until processing. Horizontal sections of 15 μm thickness were prepared, using the cryostat (Bright OTF Cryostat, Bright Instrument Company Ltd., Huntington, UK). The sections were mounted on glass slides that had been previously gelatinized and were stored at −20°C until use for immunohistochemistry. Double immunostaining was performed using a goat polyclonal antibody against Brn3a (see above), and a rabbit polyclonal antibody against PKCα, expressed in rod bipolar cells (1/1000; P4334, Sigma-Aldrich); alternatively, a mouse monoclonal antibody against Syt2 (1/100; AB10013783, Zebrafish International Resource Center, University of Oregon, Eugene, OR, USA), and a rabbit polyclonal antibody against GNB3 (1/50, HPA005645 Sigma-Aldrich, Goettingen, Germany) were used, all diluted in 0.1M phosphate buffer containing 1% (*v*/*v*) Triton X-100 (Sigma). DAPI (Sigma-Aldrich) was included to stain the cell nuclei. As blocking mixture, normal donkey serum was used. Immunohistochemistry negative controls were conducted in parallel, omitting each one of the primary antibodies.

After several washes with PBS, the retinas were incubated for 2 h at room temperature with Cy2 (FITC) donkey anti-goat or donkey anti-rabbit (1/200, Jackson immunoreseach Europe) and Cy3 donkey anti-rabbit secondary antibodies (1/200, Jackson immunoreseach Europe). Then, the slides were cover-slipped with anti-fading mounting medium (Citifluor Ltd.) and sealed with nail polish. The samples were observed by confocal microscopy of the Leica brand, model TCS SP2 (Leica, Wetzlar, Germany). A 40× and a 63× oil immersion lenses were used and Z projections of maximum amplitude were made to cover all the cells studied.

### 4.7. Anterograde Labeling of the Visual Pathway 

Cholera toxin based dyes were used as anterograde tracers: Cholera toxin subunit B (recombinant) Alexa Fluor® 488 conjugate (C-34775, Invitrogen, Thermo Fisher Scientific, Cambridge, MA, USA) (labeled in green) and cholera toxin subunit B (recombinant) Alexa Fluor® 555 conjugate (C-34776, Invitrogen, Thermo Fisher Scientific) (labeled in red), both at a concentration of 2% and dissolved in 0.1M PBS and 1% DMSO (dimethyl sulfoxide). The labeling was performed by intraocular injection, following the same protocol used for the excitotoxic agents. One microliter of CTX488 in the right eye and the same volume of CTX555 in the left eye. Nine days after the intraocular injection of the NMDA/KA agents, just after finishing ERG recordings, cholera toxins were injected, and after 5 days of trace labeling, perfusion, extraction and fixation of the brain was carried out for the histological study of target projection.

### 4.8. Statistical Analysis

Statistical analysis was performed using the Mann-Whitney test for nonparametric distributions, and the Student’s t-test for normal distributions. They were performed using GraphPad Prism version 5.00 for Windows (www.graphpad.com, GraphPad Software, San Diego, CA, USA).

## 5. Conclusions

This work demonstrates that the intraocular injection of glutamate agonists NMDA and KA have a deleterious effect on the inner and middle retina cells, leading to the death of bipolar, amacrine and ganglion cells and a huge reduction of the inner retinal thickness. All these morphological alterations are reflected in their functionality, as shown by the ERG recordings. In addition, there is a loss of connection with its projection cores and pathways. Our work confirms the usefulness of this animal model for the study of deleterious excitotoxicity of glutamate acting on different types of glutamate receptors.

## Figures and Tables

**Table 1:** RGC densities estimated after the individual administration of KA and NMDA, and the joint administration of NMDA/KA. RGC densities are expressed as arithmetic mean and standard deviation (DS). The number of animals used in each case is indicated, as well as the statistical significance (*p*) of the comparative analysis between the control left eye (LE) and the experimental right eye (RE) (Student’s *t* test).

	KA			NMDA			NMDA/KA
	5 mM	10 mM	20 mM	10 mM	30 mM	100 mM	30/10
**LE**	2431 (198)	2206 (229)	2102 (631)	2789 (325)	2433 (308)	2761 (302)	2372 (133)
**RE**	1615 (435)	1133 (410)	734 (428)	2075 (853)	2008 (403)	1229 (457)	1060 (209)
***p***	0.17	0.02	0.01	0.5	0.19	0.03	0.002
***n***	4	4	3	3	4	4	7
